# Current training landscape for novice robotic surgeons: an international investigative survey by the Junior-ERUS/Young academic urologists (YAU) robotics in urology working group

**DOI:** 10.1007/s00345-025-05845-5

**Published:** 2025-08-01

**Authors:** Christoph Würnschimmel, Mike Wenzel, Marcio Covas Moschovas, Paolo Dell’Oglio, Marco Paciotti, Carlo Andrea Bravi, Ruben De Groote, Fabrizio Di Maida, Federico Piramide, Filippo Turri, Iulia Andras, Gabriele Sorce, Nikolaos Liakos, Alberto Breda, Alessandro Larcher

**Affiliations:** 1 Department of Urology, Luzerner Kantonsspital, Lucerne, Switzerland; 2 Faculty of Health Sciences and Medicine, University of Lucerne, Lucerne, Switzerland; 3 Department of Urology, University Hospital Frankfurt, Goethe University Frankfurt, Frankfurt am Main, Germany; 4 Global Robotics Institute, AdventHealth, Celebration, FL USA; 5 Department of Urology, ASST Niguarda Hospital, Milan, Italy; 6 Department of Urology, IRCCS Humanitas Research Hospital, Rozzano, Milan, Italy; 7 Department of Urology, Northampton General Hospital, Northampton, UK; 8 Department of Urology, The Royal Marsden NHS Foundation Trust, London, UK; 9 Department of Urology, OLV Hospital, Aalst, Belgium; 10 ORSI Academy, Melle, Belgium; 11 Unit of Oncologic Minimally Invasive Urology and Andrology, Department of Experimental and Clinical Medicine, Careggi Hospital, University of Florence, Florence, Italy; 12 Division of Urology, Department of Oncology, San Luigi Gonzaga Hospital, University of Turin, Orbassano, Turin, Italy; 13 Department of Urology, ASST Santi Paolo e Carlo, University of Milan, Milan, Italy; 14 Department of Urology, Iuliu Hatieganu University of Medicine and Pharmacy, Cluj- Napoca, Romania; 15 Division of Experimental Oncology, Department of Urology, Urological Research Institute (URI), IRCCS San Raffaele Scientific Institute, Milan, Italy; 16 Department of Urology, Faculty of Medicine, Medical Centre of the University of Freiburg, Freiburg, Germany; 17 Department of Urology, Fundació Puigvert, Autonomous University of Barcelona, Barcelona, Spain

**Keywords:** Robotic education, Simulator, Dry-lab, Wet-lab, Robotic surgery, Survey

## Abstract

**Introduction:**

While robotic surgical training is crucial for preparing skilled surgeons, the landscape of available training programs is not well-defined. Many institutions offer structured curricula, yet transparency about training modalities, caseloads, and eligibility criteria for novice surgeons is limited. To address this gap, a structured survey was designed to assess robotic education offerings globally.

**Patients and methods:**

A web-based survey was distributed to different robotic societies, institutions and dedicated robotic surgery experts, based on the Junior European Association of Urology Robotic Section (J-ERUS) network and the Young Academic Urologists (YAU) Robotic Section between February and September 2024. Furthermore, a peer-esteem snowballing approach allowed the survey to expand its reach through expert referrals. The survey captured information on training modalities, infrastructure, caseload, and case mix. Respondents were required to provide contact details for further follow-up, while their identities and institutions remained confidential.

**Results:**

The survey achieved a 16.5% response rate, with 80 respondents from 49 institutions confirming robotic training opportunities. Training platforms included Da Vinci multi-port systems (71%), HUGO-RAS (15%), and Versius (8%). Training methods featured simulators (89%), dual-console training (65%), dry-labs (39%), and wet-labs (16%). Variability in training structures was observed, with 32% of institutions offering dedicated fellowships and 68% combining training with clinical duties. Institutions varied in case volumes (100–500 cases per year), and 41% indicated performing over 500 robotic procedures annually. Respondents predominantly answered that robotic surgery novices may access about 20% of these cases.

**Conclusion:**

This study highlights the heterogeneity of robotic surgical education and the need for standardized, globally accessible training frameworks. Establishing an international consortium to map training programs and content could enhance transparency and support novice surgeons in selecting institutions that align with their career goals. It is critical to integrate emerging robotic platforms and evolving methodologies into curricula to ensure comprehensive and effective training.

**Supplementary Information:**

The online version contains supplementary material available at 10.1007/s00345-025-05845-5.

## Introduction

A robotic training program, offering both adequate caseload and a structured education, is one of the cornerstones in the education of novice robotic surgeons [1]. While several renowned institutions providing these surroundings may come to mind for experts in their field, the situation is less clear for novices looking for guidance. Consequently, novice robotic surgeons would be dependent on individual expert guidance to identify centers of excellence which appear potentially suitable when seeking a robotic training program. Apart from this, the number of robotic procedures is increasing rapidly, and it is unclear, if the growing demand for robotic education could even be satisfied in the future. Also, the type of training and structure offered within many institutions is not transparent and should be addressed, in order to understand the need for potential improvement [2]. A training facility that offers an expert-guided, standardized training curriculum in a modern surrounding, including simulators and dry/wet-labs, but also adequate exposure in the operating theatre, would not only allow a more comprehensive learning, but would also offer a high quality of surgery and safety for the patients [3]. Therefore, based on prior experience and investigations specifically performed for robot-assisted radical prostatectomy [4], the need for a more generalized structured assessment of the status of robotic education in urology was discussed. This assessment would ideally be performed by the means of a comprehensive standardized survey that would include the possibility to refer other colleagues and institutions in order to expand the reach this assessment. Therefore, this survey, which could be redistributed every time new potential facilities are identified, would mark the beginning of the creation of a comprehensive overview of institutions which offer robotic education. This map could potentially guide novice robotic surgeons to identify those institutions that would be most fitting for their individual career. Therefore, the aim of this current investigation was to assess the status of robotic education in a structured way, but also to motivate the creation of a network of teaching facilities.

## Methods

A web-based survey *(“Google Docs”*, www.google.com/docs/about*)* investigated modalities and infrastructure provided for training, including the type of robotic system(s) and/or simulators provided, and the caseload and case-mix per surgeon per year (Supplementary Table 1). The online survey was drafted and tested for face-validity among the YAU robotic section and was thereafter distributed among all YAU sections for further validation, including responses and suggestions from experienced robotic surgeons but also novice robotic surgeons and patient-side assistants. Finally, the European Association of Urology Robotic Section (ERUS) was involved for further validation and to increase the quality of the survey. The survey was created in a fashion that allowed direct feedback to the contents of the survey by each respondent, and to refer to further experts in their field. This allowed to redistribute the survey to new recipients, in order to increase the reach (“peer-esteem snowballing”, [5]). Finally, using a network of robotic institutions and societies, the survey was distributed using either using direct e-mail invitations, or mailing lists from the involved associations (4). All responders were asked to include contact details including e-mail, for further questioning by our team, if necessary. The identities of respondents and institutions were collected in a secure database and remains undisclosed for data security and privacy, but the authors may offer help in guiding interested novice robotic surgeons who would be interested in being referred. The survey was open from February to September 2024.

## Results

In total, 589 individual invitations were sent out after three rounds of redistribution. After clearing out three duplicates and eight incomplete entries, we arrived at 97 evaluable respondents (response rate 16.5%). Among them, 80 respondents (82.5%) indicated robotic training possibilities within their facility. For all subsequent analyses, we relied on these 80 respondents, which originated from in total 39 European institutions (80%; Germany, Italy, France, Switzerland, United Kingdom, Belgium, Spain, Netherlands, Norway, Ireland) and 10 non-European institutions (20%; United States of America, Canada, India, Australia, Brazil, China). Of all respondents, 58% indicated to offer robotic education to applicants from outside of the institution, while the remaining 42% indicated that they would recruit only within their own institution after an evaluation period. The majority of respondents (68%) indicated to offer robotic education, but require the trainee to perform additional clinical and surgical tasks, while the remaining 32% offer a robotic fellowship fully devoted to robotic education, without requiring the trainee to perform any other clinical and surgical tasks.

### Training scenarios

**Fig. 1:**
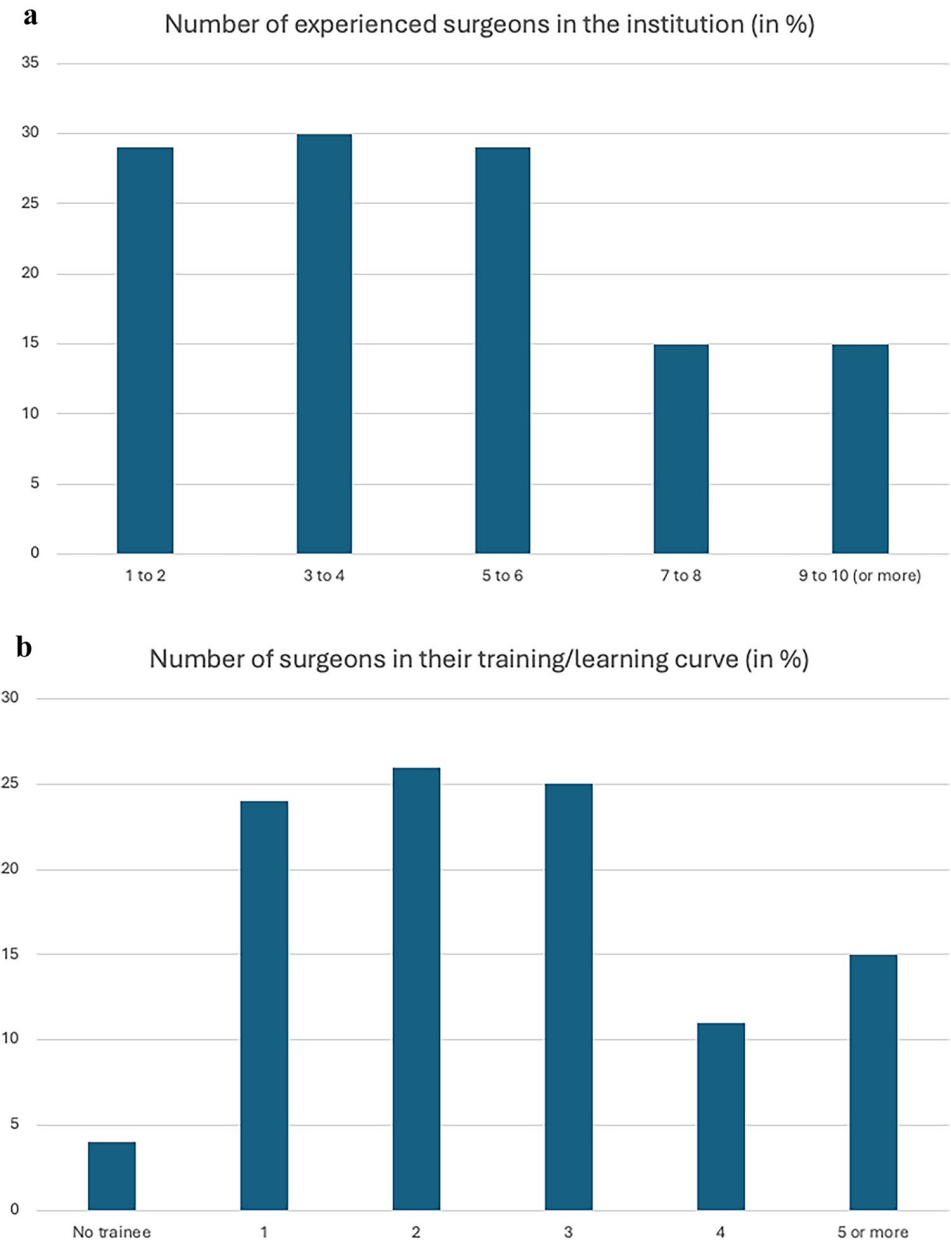
Results from an international online survey on robotic education. **a** indicates the number of experienced surgeons in the institution of the respective respondents, and **b** indicates the number of surgeons still in their robotic learning curve.

**Fig. 2:**
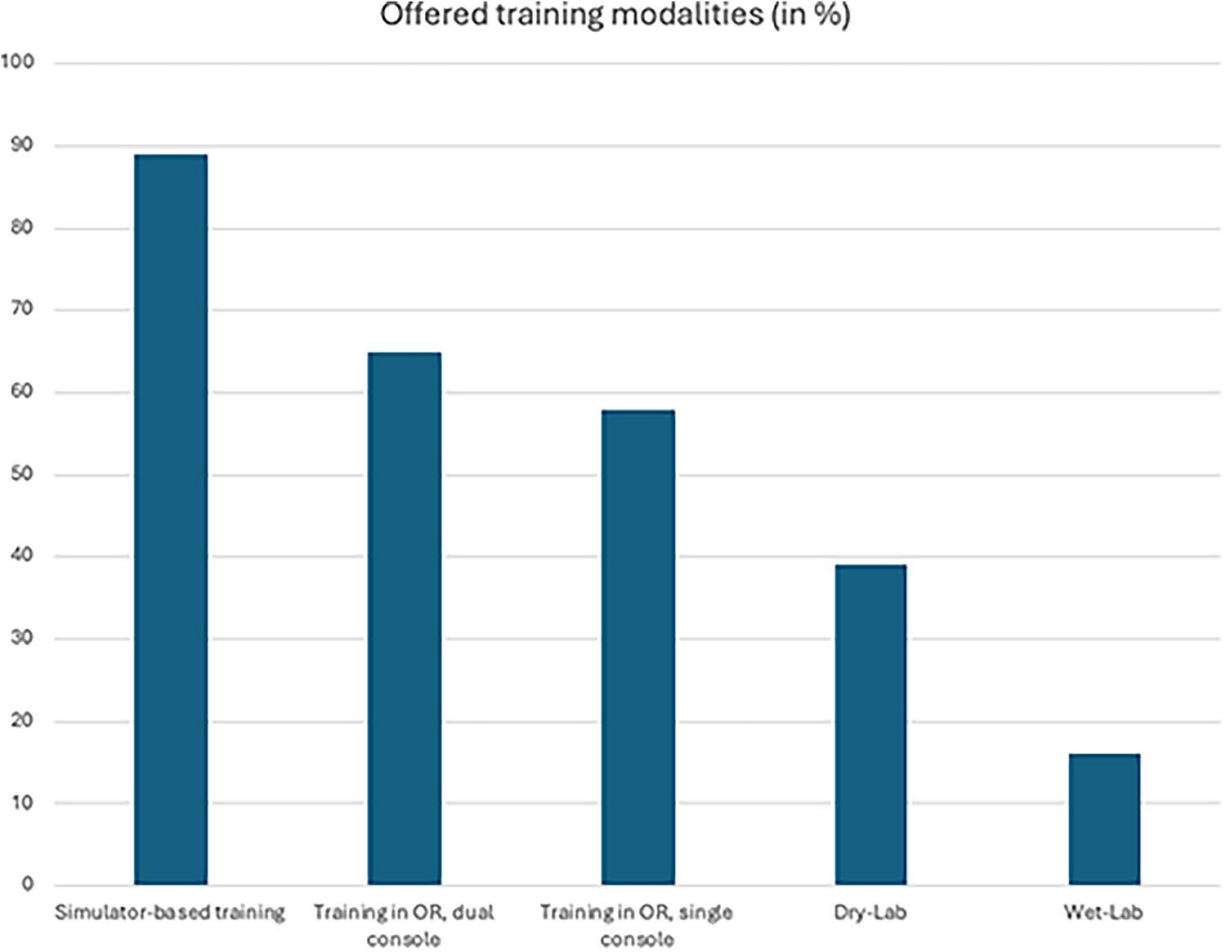
Results from an international online survey on robotic education with regard to the offered training modalities for novice robotic surgeons within the respondents’ respective institutions. OR…. Operating room

Within their institutions, 71% of respondents indicated to offer training on Da-Vinci multi-port platforms, 6% Da-Vinci single-port platforms, 15% HUGO-RAS and 8% Versius. Figure 1a and b summarize the responses stratified by the number of robotic surgeons (experts and in their learning curve) per centre. Furthermore, the majority of institutions offer simulator-based training (89%), followed by direct hands-on training within the operating theatre during surgeries, using a dual-console setup (65%), or a single-console setup (58%), depending on situative availability (Fig. 2). Furthermore, 39% of respondents offer a dry-lab setup to improve surgical skills, and 16% of institutions also offer a wet-lab setup.

### Case-load and case-mix for robotic education

**Fig. 3:**
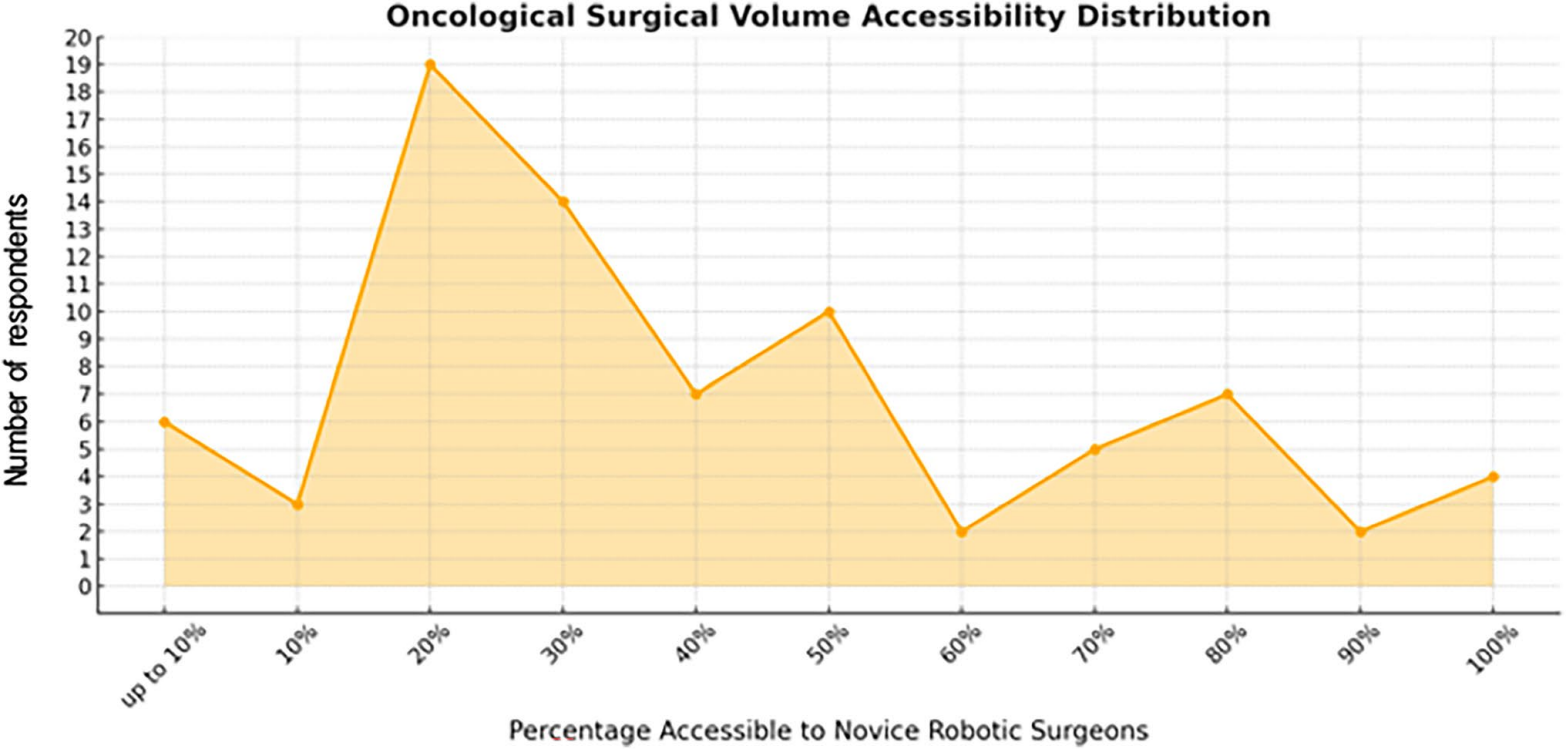
Results from an international online survey on robotic education with regard to the *oncological* surgical volume of an institution that would be accessible to novice robotic surgeons during training

**Fig. 4:**
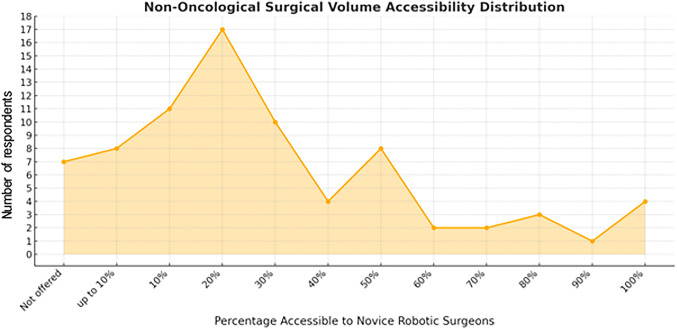
Results from an international online survey on robotic education with regard to the *non-oncological* surgical volume of an institution that would be accessible to novice robotic surgeons during training

Most respondents (41%) indicated that more than 500 robotic cases were performed in their institution per year, whereas 11%, 16%, 18% and 14% of responders had 400–500, 300–400, 200–300 and 100–200 cases performed every year, respectively. No respondent indicated a yearly caseload lower than 100 cases. Further questions included the case-mix of oncological and non-oncological procedures, that would be accessible for robotic training. Here, for oncological and non-oncological cases, the distribution of accessible cases (out of all cases performed in the institution) for novice robotic surgeons peaks around 20–30% (Fig. 3) and 10–20% (Fig. 4), respectively. Full access (100%) was indicated by four respondents (5% of all respondents), for both oncological and non-oncological cases.

## Discussion

The landscape and demands for robotic surgery training has drastically changed in the last years. With more simulator-based training possibilities available, and options to increase surgical skills in various dry-lab and wet-lab scenarios, several training curricula were established [6–8]. Furthermore, also during surgery in the OR, new technical advantages have been introduced and also the methodology of training has evolved, now including technical options such as dual console training and approaches like proficiency-based progression training within clearly structured robotic training curricula, which ultimately increased safety and efficacy of robotic training [3, 9–14]. At the same time, the annually increasing caseload in all institutions and also newly introduced robotic systems require to seek pathways to establish a proper educated offspring in robotic surgery [15–18]. In this regard, the YAU– Robotics in Urology Working Group/J-ERUS identified the need to assess the status of robotic education, in order to lay the groundwork for further improvement. As a result of discussions, based on the views from members of different countries and different health-care systems as well as different approaches to receive robotic training, the working groups have identified potential aspects that should be addressed within a structured assessment of the status quo.

One of the major aspects to address was to create a survey among expert surgeons and facilities, including the option to refer to further experts in the field, previously described as “peer-esteem snowballing” [5]. Using this approach, we aimed to create a network of experts that would reach potentially less visible facilities offering robotic education. In theory, this approach could be replicated multiple times and over some months or years, this peer-based survey creates an overview that could lay the foundation for novice robotic surgeons looking for robotic education. The aim of the first three rounds of this survey was to gain insights into the possibilities offered to novice robotic surgeons and yielded remarkable responses.

First, the response rate of our survey was 16.5%, which was comparable to our earlier investigations using the same survey format [4]. Naturally, we expected to receive responses rather from those respondents and institutions which offer training, than from those where no training is available. This pre-selection might have decreased the response rate. Furthermore, some respondents might have been reluctant to provide personal or institutional data using an online survey, which was also the reason why we decided to keep the identifying data of individual respondents and institutions undisclosed. However, more than half of the respondents indicated that potential robotic education would not only be accessible to those surgeons who were recruited from within the institution, but also to those who are willing to apply from outside. For this reason, we are of the opinion that an independent international consortium could take on the task to create an official and regularly updated “map” of institutions willing to contribute their data and who welcome outside applicants. This could be a major step in standardizing robotic education internationally, and simultaneously provides transparency for those applicants who could tailor their robotic education according to their needs.

Second, this investigation reveals the ongoing process of a “platform-switch” for some institutions. While most training curricula were created and validated for the Da Vinci multiport systems, we will face the increasing adaption of other robotic platforms, which will demand adjustments to the established training curricula. Also, some experts providing robotic education on the Da Vinci multiport platform, might still be in their learning curve on other platforms, which is the reason that case-load and case-mix adjusted to the robotic platform should be part of the evaluation. This is also especially important when offering training not only on simulators or lab-settings, but when doing so in real surgery. For the Da Vinci single port system, Pellegrino et al. suggested at least 30 simple prostatectomies, 70 nephrectomies and 150 radical prostatectomies before the complication rates reach a minimum [19].

Third, when considering the most favorable scenarios with regard to case-load and case-mix, a novice robotic surgeon might gain access to an institution that performs at least 500 cases per year (41% of all respondents). When distributing it according to access to oncological cases and non-oncological cases (which, according to the majority of institutions, peaked at 20% for both), the most realistic approximation would be that the novice would be able to operate on approximately 50 oncological and 50 non-oncological cases per year, which roughly translates into one oncological and one non-oncological procedure per week. Some institutions (14%) indicated also 100–200 cases per year per institution in total. Considering that some of these institutions also require novice robotic surgeons to perform other tasks than robotic surgery, it remains unclear if the benefit for the novice in terms of a quick learning curve would be present. Although it cannot be translated directly to an education setting, a systematic review by Van den Broeck et al. indicated that surgical volume might indeed be important in order to expect better outcomes [20].

In conclusion, the current investigation points out the changing landscape of robotic surgical education and displays the heterogeneity between institutions. Especially for those institutions that may not be able to offer an adequate caseload, different approaches for robotic training methodology are emerging. Robotic surgery novices should aim to apply in institutions that provide an appropriate case-load and case-mix, complemented by simulator-based training, dry-labs and wet-labs, combined with new methodological approaches such as proficiency-based progression trainings within a structured robotic training curriculum [12, 21, 22]. Using the results of this survey, renowned registries such as the “European Fellowship Registry in Urology” maintained by the European Society of Residents in Urology, could be expanded even further and could create a prospective mainstay for fellows looking for guidance in robotic education [23].

One limitation of this investigation includes the “bona fide” assessment using an online survey. However, all respondents provided their names and contact details, which in our opinion increases the data quality. A potential selection bias may be in effect, due to the low number of respondents. Especially, according to the responses gathered, and even though this survey was executed as an international survey including all countries interested in participating, the dominant countries within this survey were from Europe (80%). Therefore, care should be given to interpret the data outside of Europe. Finally, despite several rounds of invitations, the response rate could not be significantly increased, indicating the need to identify other channels and ways to distribute the survey in future iterations (i.e. QR-Codes on international meetings).

## Conclusion

This study highlights the heterogeneity of robotic surgical education and the need for standardized, globally accessible training frameworks. Many institutions appear to provide a capable environment for teaching novice robotic surgeons, however, with varying surgical exposure. For institutions without an adequate caseload per surgeon, auxiliary measures for a comprehensive training including dry-lab and wet-lab trainings and reliable educational tools such as proficiency-based-progression methodology may be considered. Establishing an international consortium to map training programs and content could enhance transparency and support novice surgeons in selecting institutions that align with their career goals. Emerging robotic platforms and evolving methodologies must also be integrated into curricula to ensure comprehensive and effective training.

## Supplementary Information

Below is the link to the electronic supplementary material.

Supplementary Material 1

## Data Availability

Data is provided within the manuscript and supplementary information files.
