# Supplementary material for: Current training landscape for novice robotic surgeons: an international investigative survey by the Junior-ERUS/Young academic urologists (YAU) robotics in urology working group
Source: World J Urol. 2025 Aug 1;43(1):467. doi: 10.1007/s00345-025-05845-5 (PMC12316765; doi:10.1007/s00345-025-05845-5)
Supplement: Supplementary file 1 — Supplementary Material 1 [file 345_2025_5845_MOESM1_ESM.docx]

**For a start, please provide your name, institution, position in your institution and country.**

1. How many experienced surgeons regularly perform robotic surgery in your institution?
2. How many surgeons are currently in their learning curve for robotic surgery in your institution?
3. Do you offer robotic education for novice robotic surgeons in your institution?
   Please choose the response that fits best, or provide a free response. 
   If necessary, more than one response is possible.
4. Which infrastructure and modalities for robotic education can you provide in your institution? 

   If you provide additional explicit robotic training curricula outside of the hospital environment (such as ORSI or IRCAD), please indicate these details in the separate field below.
5. What is the approximate annual volume of the following ONCOLOGICAL robotic surgeries performed at your institution?

   Please provide numbers of ONCOLOGICAL surgeries that fall into the below categories, like radical prostatectomy, radical cystectomy, partial/radical nephrectomy or nephroureterectomy. A free text response is also possible.
6. Of the above ONCOLOGICAL surgical volume performed at your institution, what is the approximate percentage that is accessible for novice robotic surgeons in a supervised teaching-scenario?
7. What is the approximate annual volume of the following NON-ONCOLOGICAL robotic surgeries performed at your institution?

   Please provide numbers of NON-ONCOLOGICAL surgeries that fall into the below categories, like prostate adenomectomy, resection of strictures, ureteral re-implantations or ureteral replacement, bladder augmentation, pyeloplasty etc. A free text response is also possible.
8. Of the above NON-ONCOLOGICAL surgical volume performed at your institution, what is the approximate percentage that is accessible for novice robotic surgeons in a supervised teaching-scenario?
9. Which robotic platforms do you provide for robotic education in your institution? Free text responses possible.
10. Who was the mentor for your own robotic education?

    Please provide the name of your mentor(s) and robotic-surgery institution(s) you were educated in (in case of multiple mentors and institutions, please simply separate using semicolons).
11. Which mentor and facility outside of your institution, but within your own country would you personally recommend for novice robotic surgeons?

    If multiple, please separate using semicolons.
12. Which mentor and facility outside of your institution, worldwide would you recommend for novice robotic surgeons?

    If multiple, please separate using semicolons.
13. Do you have any additional feedback on the questionnaire?
14. Is your institution certified by an official society for robotic surgery? (for example ERUS; see https://myeau.uroweb.org/myeau/robotic-host-centre) 
    If yes, please indicate the type of certification in the separate field below.
